# Acute Effect of Betel Quid Chewing on Brain Network Dynamics: A Resting-State Functional Magnetic Resonance Imaging Study

**DOI:** 10.3389/fpsyt.2021.701420

**Published:** 2021-08-24

**Authors:** Xiaojun Huang, Zhipeng Wu, Zhening Liu, Dayi Liu, Danqing Huang, Yicheng Long

**Affiliations:** ^1^National Clinical Research Center for Mental Disorders, and Department of Psychiatry, The Second Xiangya Hospital, Central South University, Changsha, China; ^2^Department of Clinical Psychology, Jiangxi Provincial People's Hospital Affiliated to Nanchang University, Nanchang, China

**Keywords:** betel quid, addiction, substance dependence, fMRI, dynamic functional connectivity, dynamic brain network

## Abstract

Betel quid (BQ) is one of the most popular addictive substances in the world. However, the neurophysiological mechanism underlying BQ addiction remains unclear. This study aimed to investigate whether and how BQ chewing would affect brain function in the framework of a dynamic brain network model. Resting-state functional magnetic resonance imaging scans were collected from 24 male BQ-dependent individuals and 26 male non-addictive healthy individuals before and promptly after chewing BQ. Switching rate, a measure of temporal stability of functional brain networks, was calculated at both global and local levels for each scan. The results showed that BQ-dependent and healthy groups did not significantly differ on switching rate before BQ chewing (*F* = 0.784, *p* = 0.381, analysis of covariance controlling for age, education, and head motion). After chewing BQ, both BQ-dependent (*t* = 2.674, *p* = 0.014, paired *t*-test) and healthy (*t* = 2.313, *p* = 0.029, paired *t*-test) individuals showed a significantly increased global switching rate compared to those before chewing BQ. Significant corresponding local-level effects were observed within the occipital areas for both groups, and within the cingulo-opercular, fronto-parietal, and cerebellum regions for BQ-dependent individuals. Moreover, in BQ-dependent individuals, switching rate was significantly correlated with the severity of BQ addiction assessed by the Betel Quid Dependence Scale scores (Spearman's rho = 0.471, *p* = 0.020) before BQ chewing. Our study provides preliminary evidence for the acute effects of BQ chewing on brain functional dynamism. These findings may provide insights into the neural mechanisms of substance addictions.

## Introduction

Betel quid (BQ) is an addictive substance made from areca nut, which is chewed by ~600 million people worldwide, mostly in Asia (1, 2). It ranks the fourth most popular psychoactive substance globally, following only alcohol, nicotine, and caffeine (3, 4). Nowadays, BQ addiction has become a notable public health issue not only for its high prevalence especially in parts of Asia (5, 6), but also for the increasing awareness of its relationship with a number of serious diseases such as periodontitis (7) and oral cancer (8). Nevertheless, the neurophysiological mechanism of BQ addiction are still unclear (9).

Resting-state functional connectivity (FC) which is calculated based on functional magnetic resonance imaging (fMRI) has become a major method to study functional organization of human brains (10–12). In recent years, there has been growing interest in exploring the effects of BQ chewing on brain function in terms of changed FC patterns. For example, a number of studies have reported that BQ-dependent chewers show altered FC patterns compared with non-addictive normal controls (4, 13–17), which widely involve the reward, impulsivity and cognitive systems in the brain (18). Moreover, by comparing the brain FC before and after BQ chewing, an acute effect of BQ chewing on FC patterns has been proved in both BQ-dependent chewers (9) and normal controls (19, 20). Appreciably, these findings have significantly advanced our understanding of the neural mechanism underlying BQ addiction.

All the above studies were performed under the conventional assumption that brain's FC patterns are consistent during the whole fMRI scan. However, there is a growing body of evidence supporting that FC patterns in fact fluctuate over time, which is ignored by conventional “static” analysis methods (21, 22). Thus, the “dynamic FC” has become a new hot spot in neuroimaging studies, which have been associated with learning (23), cognition (24), emotion (25), personality traits (26, 27), as well as common psychiatric diseases including schizophrenia (28), major depressive disorder (29) and bipolar disorder (30). In particular, it was suggested that using addictive substances such as alcohol (31), nicotine (32), cannabis (33), and cocaine (34) could alter the dynamic FC patterns. For example, it was found that patients with alcohol use disorder exhibited significantly higher temporal variability of FC between the cerebellum and both the frontoparietal and ventral attention networks (31). These evidences bring the possibility that dynamic FC may be affected by BQ chewing, which remains an open question to date. New contributions would thus be made by exploring dynamic FC, which focuses on the temporal stability rather than static connectivity strength in functional brain network, to understanding the pathological mechanism of BQ dependence.

In this study, therefore, we aimed to investigate the possible effects of BQ chewing on brain function as characterized by dynamic FC for the first time. Specifically, a multilayer dynamic brain network model was used and network dynamics were evaluated by its switching rate (35, 36) or “flexibility” in other words (23, 37), which is a validated metric to quantify the temporal stability of network modular structures. Both the chronic and acute effects of BQ chewing on switching rates were then investigated based on our hypotheses that (1) chronic effects of BQ chewing would be reflected by differences between healthy and BQ-dependent individuals; and (2) acute effects of BQ chewing would be shown by a comparison between before- and after-BQ chewing scans. It was anticipated that our results would be complementary to previous findings in conventional static FC-based studies, and further improve our understanding of BQ and other substance addictions.

## Materials and Methods

### Participants and Assessments

A total of twenty-four male BQ-dependent individuals were recruited from the Changsha city, Mainland China based on the following inclusion criteria: (1) 18–40 years of age; (2) right-handed; (3) Han Chinese ethnicity; (4) meeting the diagnostic criteria for BQ dependence based on the Betel Quid Dependence Scale (BQDS) (BQDS total score > 4), which was proved to be suitable for measuring BQ dependence in Chinese speakers (38). The exclusion criteria include: (1) any history of diagnosed psychiatric disorders or other severe physical illnesses; (2) any substance abuse history other than BQ; (3) any contraindication to fMRI scanning. Twenty-six age- and sex-matched non-addictive healthy controls (HCs) were also recruited from a university in Changsha area based on the same inclusion and exclusion criteria, except that they did not meet the diagnostic criteria for BQ dependence. All participants were asked to not use BQ or any other psychoactive substance during the 24 h before the experiment. The study was approved by the Ethics Committee of Second Xiangya Hospital, Central South University in Changsha, and written informed consent was obtained from all individuals.

All BQ-dependent individuals reported their duration of BQ use and completed the BQDS to assess the severity of BQ addiction. The BQDS is a 16-item self-administered scale with each item employing a dichotomous outcome (“no” = 0 and “yes” = 1), which was validated in both Chinese- (38) and English-language (39) contexts. All participants also completed the Beck Depression Inventory (BDI) (40) and Beck Anxiety Inventory (BAI) (41) to assess their emotional states before fMRI scanning, as BQ dependence is known to be related to negative emotions such as depression (42).

### Imaging Data Acquisition and Preprocessing

For each participant, resting-state fMRI scans were acquired twice (before and after BQ chewing) using a 3.0 T Philips scanner with the following parameters: matrix = 64 × 64, slices = 36, slice thickness = 4 mm, gap = 0 mm, flip angle = 90°, field of view (FOV) = 240 × 240 mm^2^, repetition time (TR) =2,000 ms, echo time (TE) = 30 ms, and total volumes = 250. After the first fMRI scan, the participants were instructed to chew the BQ and swallow the saliva as soon as possible within 3 min. The second fMRI scan was started after another 3 min. Before the first fMRI scan, T1-weighted high-resolution structural images were also acquired to aid in registration with the following parameters: matrix = 256 × 200, slices = 180, slice thickness = 1 mm, gap = 0 mm, flip angle = 8°, FOV = 240 × 240 mm^2^, TR = 7.5 ms, and TE = 3.7 ms. More details regarding the data acquisition parameters of this scanner can be found elsewhere (43, 44).

Imaging data preprocessing was performed using the DPARSF software (http://rfmri.org/DPARSF) (45, 46) and a standard pipeline whose details can be found elsewhere (29, 47). Briefly, it includes removing the first 10 volumes, slice timing, motion realignment, spatial normalization, temporal filtering (0.01–0.10 Hz), and regressing out for the white matter, cerebrospinal fluid and global mean signals. We performed global signal regression (GSR) here based on the suggestion that GSR is important to diminish motion artifacts in dynamic FC analyses (48, 49). All acquired images was manually checked by trained researchers after preprocessing to ensure the quality. None of the participants had overt artifacts or severe head motions (rotations > 2° or translations > 2 mm) for any scan, and no difference was detected in head motion measured by mean framewise-displacement (FD) (50) between the before- and after-BQ chewing fMRI scans for any group (both *p* > 0.05).

### Switching Rates of Dynamic Brain Network

After preprocessing, the switching rates of functional brain networks were calculated for each fMRI scan in the framework of a multilayer dynamic brain network model. The calculation strictly followed previously published studies (26, 28, 35, 51–54), and is summarized as follows:

1. Constructing multilayer brain network: the mean time series of 160 brain regions (nodes) defined by the Dosenbach atlas (55) were firstly extracted and segmented into continuous time windows using a sliding-window approach, which is one of the simplest and most commonly used methods to construct dynamic brain networks (29, 35, 56). Based on prior recommendations that the window width should exceed the inverse of the slowest frequency component in the signals (57, 58), a window width of 50 volumes (100 s) and a step length of one volume (2 s) were used, resulting in a total of 191 windows. Within each window, the FC strengths between all pairs of nodes were estimated by Fisher's z-transformed Pearson correlation coefficients, yielding a 160 × 160 symmetric connectivity matrix. These time-ordered matrices formed a dynamic brain network *G* = (*G*_*t*_)_t = 1, 2, 3, …, 191_, where the *t*th connectivity matrix (*G*_*t*_) represents the “snapshot” of brain network organization within the *t*th window.
2. Detecting time-varying modular structures: a multilayer modularity algorithm described by Mucha et al. (59) was implemented to detect the time-varying modular structures of brain network within each time window. This was achieved using an open-source Matlab-based code package (https://github.com/GenLouvain/GenLouvain) (60) with the default settings. In line with previous studies, all negative values in the connectivity matrices were set to zeros before applying the algorithm (26, 35, 61). As the output, a 160 (number of nodes) × 191 (number of time windows) module assignment matrix was obtained for each fMRI scan, representing the temporal alterations in module assignments for all the 160 nodes.
3. Calculating network switching rates: the switching rate for a node *i* (*f*_*i*_) was calculated as $f_{i}=\frac{n_{i}}{N}$ based on the above module assignment matrices, where *n*_*i*_ is the number of times it “switched” from one module to another module, and *N* is the maximum potential number of switch (equaled to 191–1 = 190 here). The calculation was performed using the Network Community Toolbox (http://commdetect.weebly.com) (23). Switching rates for the whole brain and six large-scale subnetworks (default-mode, occipital, cingulo-opercular, fronto-parietal, sensorimotor, and cerebellar) as defined by Dosenbach et al. (55) were then obtained by averaging all the 160 nodes or the nodes of each subnetwork, respectively (26, 28). The values of switching rates at all levels can range from 0 to 1 theoretically, with a higher switching rate indicating a lower temporal stability of brain networks. Notably, the whole process was repeated for 100 times and averaged to get the final results, since slightly different results can be produced during each modular detection (26, 51, 52, 62).

### Statistics

Differences in demographic and clinical characteristics between HCs and BQ-dependent individuals were tested using two-sample *t* tests. Possible effects of BQ chewing on brain network switching rate were investigated with the following procedures: first, to investigate if there is a significant chronic effect of BQ dependence, group difference in switching rates between HCs and BQ-dependent individuals before BQ chewing was tested using analysis of covariance (ANCOVA), with age, years of education, and head motion measured by mean FD as covariates; second, to investigate if there is an acute effect of BQ chewing for all participants despite the histories of BQ use, and if it would differ across healthy and BQ-dependent groups, switching rates before and after chewing BQ were compared using paired *t*-tests for the entire sample with *post-hoc* comparisons for each group separately. Relationships between the switching rates and clinical characteristics (BQDS, BDI, and BAI scores, as well as duration of BQ use) were also assessed using Spearman correlations. All statistics on switching rates were performed at the global, sub-network, and nodal levels. Significance was set at *p* < 0.05 and False discovery rate (FDR) corrections (63) were performed to control for type I errors across the 6 sub-networks/160 nodes. The results were visualized by the BrainNet Viewer (64).

## Results

### Sample Characteristics

Demographic and clinical characteristics of the participants are summarized in Table 1. There was no significant difference in age between the BQ-dependent and healthy individuals (*t* = −1.175, *p* = 0.246). The BQ-dependent individuals had significantly less years of education (*t* = −2.482, *p* = 0.021), higher BDI score (*t* = 4.044, *p* < 0.001) and higher BAI score (*t* = 3.818, *p* = 0.001) than healthy individuals.

**Table 1:** Demographic and clinical characteristics of the participants.

	**BQ-dependent individuals (*n* = 24), mean ± SD**	**Healthy controls (*n* = 26), mean ± SD**	**Group comparisons**
Age (years)	23.54 ± 3.87	24.50 ± 1.48	*t* = −1.175, *p* = 0.246
Gender (male/female)	24/0	26/0	*/*
Education (years)	15.13 ± 1.73	16.00 ± 0.00	*t* = −2.482, *p* = 0.021
Duration of BQ use (years)	7.75 ± 4.28	/	*/*
BQDS score	7.42 ± 1.86	/	*/*
BDI score	10.58 ± 6.68	4.04 ± 4.65	*t* = 4.044, *p* < 0.001
BAI score	28.50 ± 6.20	23.27 ± 2.68	*t* = 3.818, *p* = 0.001

### Effects of BQ Chewing on Switching Rates

**Figure 1:**
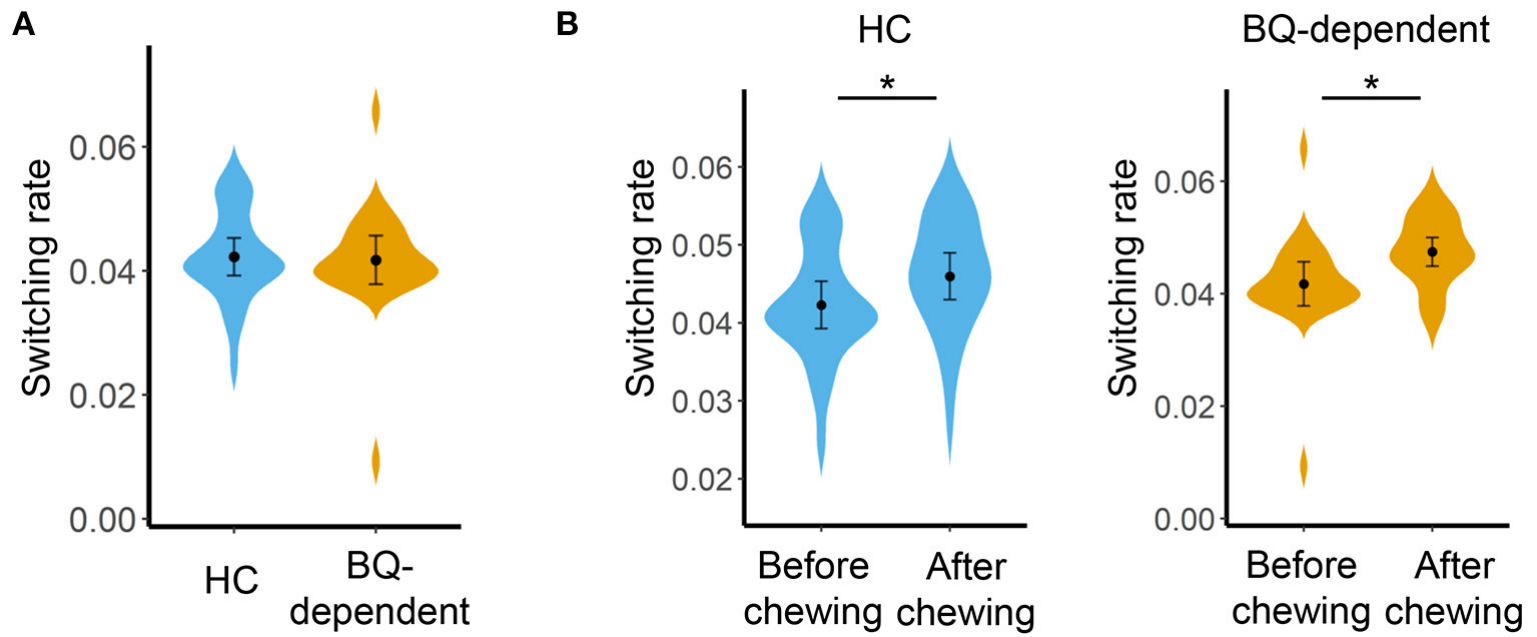
**(A)** Comparisons on global switching rate between the HC and BQ-dependent groups before BQ chewing. **(B)** Comparisons on global switching rate between the before- and after-BQ chewing scans for each group. The “*” indicates a significant difference with *p* < 0.05. BQ, betel quid; HC, healthy controls.

Before BQ chewing, there was no significant difference in global switching rate between the HC and BQ-dependent groups (*F* = 0.784, *p* = 0.381, as shown in Figure 1A); no significant groups differences were observed at the sub-network- or nodal levels (all corrected-*p* > 0.05), either.

**Figure 2:**
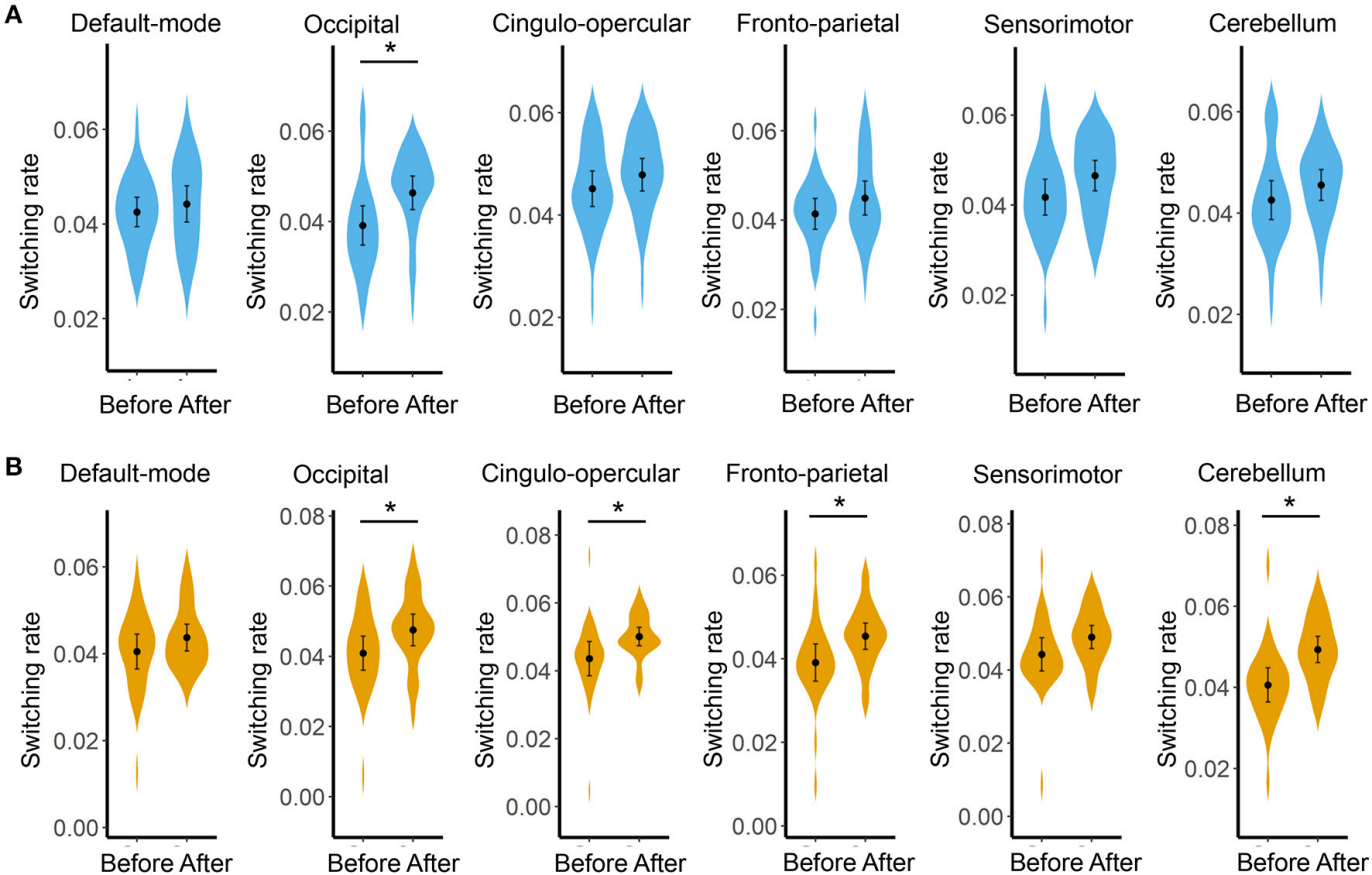
Results of comparisons on the switching rate of each subnetwork between the before- and after- BQ chewing scans for each group. **(A)** Results for the healthy controls. **(B)** Results for the BQ-dependent individuals. The “*” indicates a significant difference with *p* < 0.05.

**Figure 3:**
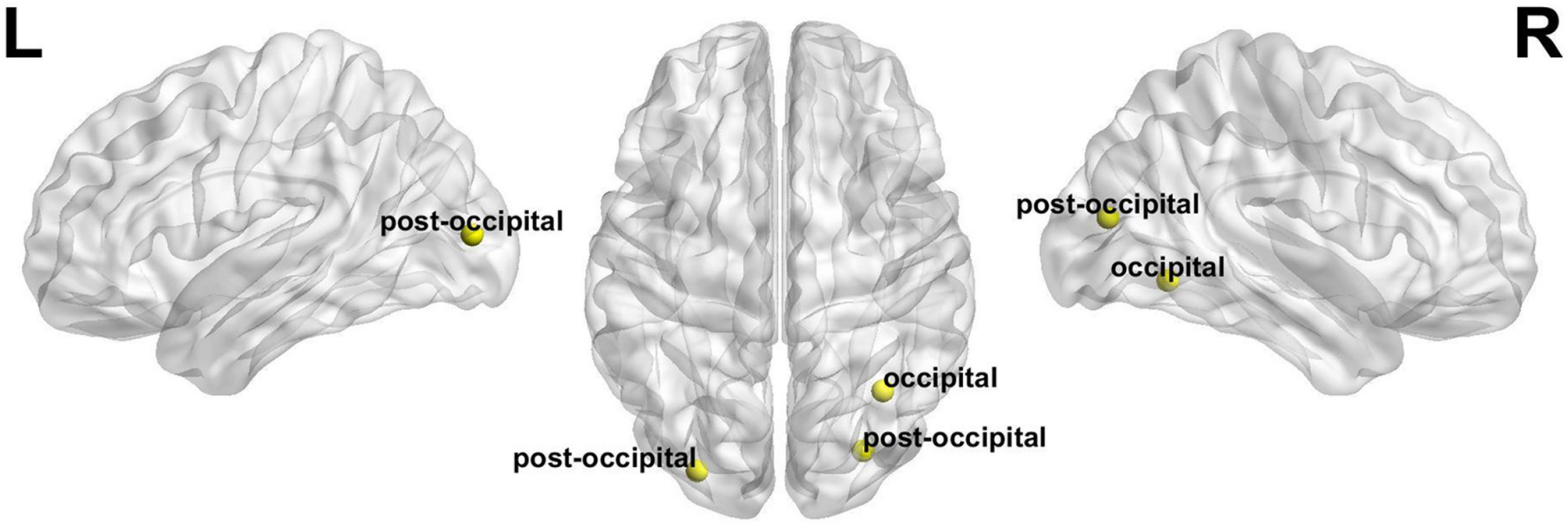
The nodes whose switching rates were found to be significantly increased after BQ chewing in both the healthy and BQ-dependent individuals (all with *p* < 0.05). “L” and “R” refer to “left” and “right”, respectively.

Compared with before BQ chewing, a significantly increased global switching rate was found for all participants after BQ chewing when tested within the entire sample (*t* = 3.549, *p* = 0.001). *Post-hoc* comparisons showed that both the HCs and BQ-dependent individuals showed a significantly increased global switching rate after BQ chewing (*t* = 2.313, *p* = 0.029 and *t* = 2.674, *p* = 0.014 for HCs and BQ-dependent individuals, respectively; shown in Figure 1B). At subnetwork and nodal levels, significantly increased switching rates were found within the occipital, cingulo-opercular, fronto-parietal, and cerebellum areas (all corrected-*p* < 0.05, see Supplementary Tables 1, 2); *post-hoc* comparisons showed that both the HCs and BQ-dependent individuals had increased switching rates within the occipital area, while significantly increased switching rates within the cingulo-opercular, fronto-parietal and cerebellum subnetworks were found in only the BQ-dependent group (Figures 2, 3, and see details in Supplementary Tables 1, 2).

### Correlations

**Figure 4:**
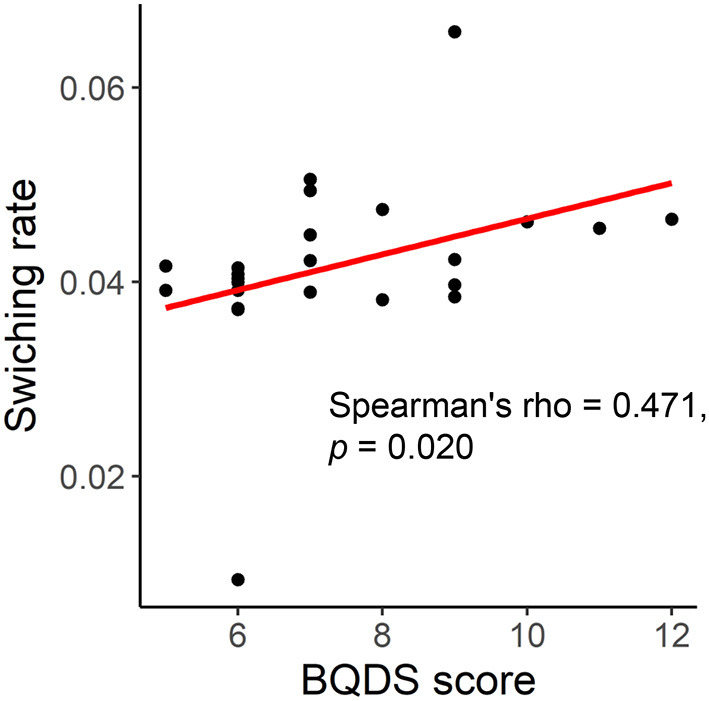
Correlation between the global switching rate and Betel Quid Dependence Scale (BQDS) score in the BQ-dependent individuals.

Before BQ chewing, a significant correlation was found between the global switching rate and BQDS score in the BQ-dependent individuals (Spearman's rho = 0.471, *p* = 0.020, Figure 4). However, such a correlation became not significant when performing a partial correlation controlling for age, years of education and head motion (Pearson's *r* = 0.355, *p* = 0.114). No significant correlations were found for any other scales, or at the local levels (all corrected-*p* > 0.05).

## Discussion

In this study, we investigated the effects of BQ chewing on brain function in the framework of a dynamic brain network model. The results showed that after BQ chewing, both the BQ-dependent and healthy individuals showed a significantly increased switching rate in their brain networks. Such results may thus provide evidence for an acute effect of BQ chewing on brain functional dynamism.

As one of the most popular addictive substances worldwide, BQ has been proved to have acute effects on brain function which can be revealed by fMRI (9, 19, 20). These effects include, for example, significantly increased FC within the subcortical and visual cortical regions which was thought to relate to acutely rewarding and visual effects produced by arecoline (19). However, all previous studies were performed under the assumption that brain's FC patterns are static, in which the important details regarding temporal fluctuations of FC would be ignored (21, 22). The present study, as far as we know, is the first to focus on BQ-related effects on the stability of dynamic FC.

We found that after BQ chewing, all participants showed an increased global switching rate (Figure 1B) which suggests a temporally more variable (less stable) functional brain network organization (26). Although not completely clear, temporal variability of FC is thought to reflect a general readiness for reorganizing the brain network in response to changing attentional and cognitive demands (31, 51, 65, 66). Meanwhile, excessively increased temporal variability of FC during rest has been reported in multiple diseases including schizophrenia (28), major depressive disorder (29), bipolar disorder (30), and autism spectrum disorder (67), potentially reflecting a under-constrained brain network and disrupted information exchanges among brain systems (26, 31, 35). Interestingly, some other recent studies have reported that the uses of alcohol (31), nicotine (32), and cannabis (33) are all related to an increased temporal variability of functional brain network. Combining these previous findings with our results, it may be considered that increased temporal variability of FC is a common hallmark underlying the development of multiple substance use disorders including BQ dependence.

At local level, both the healthy and BQ-dependent groups exhibited significantly increased switching rates within the occipital areas (Figures 2, 3). The occipital cortex is known to be the visual processing center of human brain (68), and may also be involved in the processing of memory (68) and emotions (69) as indicated by some reports. Interestingly, an increased activation in occipital cortex has been consistently observed in drug users when in response to drug-related cues, even for non-visual drug-related stimuli (70–72). Increased FC within the visual cortex has also been observed after acute BQ (19) and alcohol (73) consumptions as well as in cocaine-dependent individuals (74). Here, our results thus further highlight the important role of occipital/visual cortex in the effects of addictive substance in the context of dynamic FC. In the BQ-dependent individuals, significantly increased switching rates after BQ chewing were also found within the cingulo-opercular, fronto-parietal, and cerebellum subnetworks (Figure 2). Such results are in line with multiple previous static fMRI studies, which reported effects of BQ chewing on the same subsystems (4, 9, 20). Based on the hypothesis that excessively increased switching rates may indicate disorganized brain network dynamics (53), the above alterations may be reflective of a neural reorganization of cerebral-cerebellum functional network caused by BQ. Meanwhile, although not significant, trend-level increases in switching rates of these sub-networks were found in HCs (Figure 2); here, we propose that similar effects may exist in HCs but are too weak to survive the statistical test.

It is noteworthy that a number of previous studies have reported chronic effects of BQ dependence on brain network, as characterized by altered static FC patterns in BQ-dependent chewers compared with HCs even when without BQ chewing (4, 13–17). However, no significant differences in switching rate were found between the BQ-dependent individuals and HCs before BQ chewing in this study (Figure 1A). Meanwhile, although a positive correlation was shown between the switching rate and BQ addiction severity (Figure 4), it became not significant when controlling for possible confounders including age, years of education and head motion. Future explorations with a larger sample may be needed to confirm if the impacts of BQ on dynamic FC can be reflected as chronic effects.

Our study has several limitations. First, the sample size is relatively small and as mentioned above, future studies with a larger sample and higher statistical power are needed to confirm the conclusions. Second, we only investigated the effects of BQ chewing on brain network dynamics during rest, and further studies may explore such relationship under specific tasks to further improve our knowledge on it. Third, the results may be influenced by different data preprocessing and analysis strategies, such as performing/not performing GSR and different window widths chosen in the sliding-window approach. Fourth, it should be noted that the BQ-dependent group had higher BDI and BAI scores than the healthy controls; therefore, it is possible that group differences observed in this study are partly driven by the differences in overall severity of depressive and/or anxiety symptoms. Fifth, only male BQ chewers were recruited and the study was performed with a cross-sectional design. Future studies will benefit from including female participants and from longitudinal designs.

In conclusion, in this study, we investigated the effects of BQ chewing on brain function in the framework of a dynamic brain network model. The results indicated that chewing BQ has an acute effect in both the BQ-dependent and healthy individuals to significantly decrease the temporal stability of functional brain network, which widely involved the occipital, cingulo-opercular, fronto-parietal, and cerebellum systems. Our study provides preliminary evidence for effects of BQ chewing on brain functional dynamism, which may also provide new insights into the neural mechanisms of substance addictions.

## Data Availability Statement

The original contributions presented in the study are included in the article/Supplementary Material, further inquiries can be directed to the corresponding authors.

## Ethics Statement

The studies involving human participants were reviewed and approved by Ethics Committee of Second Xiangya Hospital, Central South University. The patients/participants provided their written informed consent to participate in this study.

## Author Contributions

XH, ZL, and YL designed the study and carried out the analysis. XH and ZL contributed to the data collection. XH and YL wrote the first draft of manuscript. ZW, ZL, DL, and DH contributed to the final manuscript. All authors have read and agreed to the published version of the manuscript.

## Conflict of Interest

The authors declare that the research was conducted in the absence of any commercial or financial relationships that could be construed as a potential conflict of interest.

## Publisher's Note

All claims expressed in this article are solely those of the authors and do not necessarily represent those of their affiliated organizations, or those of the publisher, the editors and the reviewers. Any product that may be evaluated in this article, or claim that may be made by its manufacturer, is not guaranteed or endorsed by the publisher.
