# Supplementary material for: Acute Effect of Betel Quid Chewing on Brain Network Dynamics: A Resting-State Functional Magnetic Resonance Imaging Study
Source: Front Psychiatry. 2021 Aug 24;12:701420. doi: 10.3389/fpsyt.2021.701420 (PMC8421637; doi:10.3389/fpsyt.2021.701420)
Supplement: Supplementary file 1 [file Data_Sheet_1.pdf]

Supplemental Table S1. The subnetworks whose switching rates were found to be significantly increased after BQ chewing (FDR-corrected p <0.05).

Subnetwork	Results	Results of post-hoc tests
occipital	Increased after BQ chewing (t = 3.618, FDR-corrected p = 0.002)	Increased in both healthy (t = 2.702, p = 0.012) and BQ-dependent groups (t = 2.359, p = 0.027)
cingulo-opercular	Increased after BQ chewing (t = 2.848, FDR-corrected p = 0.010)	Increased in BQ-dependent group (t = 2.580, p = 0.017)
fronto-parietal	Increased after BQ chewing (t = 2.900, FDR-corrected p = 0.010)	Increased in BQ-dependent group (t = 2.209, p = 0.037)
cerebellum	Increased after BQ chewing (t = 3.586, FDR-corrected p = 0.002)	Increased in BQ-dependent group (t = 3.762, p = 0.001)

Supplemental Table S2. The nodes whose switching rates were found to be significantly increased after BQ chewing (FDR-corrected p <0.05), with Montreal Neurological Institute (MNI) coordinates reported here.

MNI x	MNI y	MNI z	Label	Results	Results of post-hoc tests
36	-60	-8	occipital	Increased after BQ chewing (t = 3.694, FDR-corrected p = 0.030)	Increased in both healthy (t = 2.352, p = 0.027) and BQ-dependent groups (t = 2.826, p = 0.010)
29	-81	14	post-occipital	Increased after BQ chewing (t = 4.217, FDR-corrected p = 0.009)	Increased in both healthy (t = 2.828, p = 0.010) and BQ-dependent groups (t = 3.112, p = 0.005)
-29	-88	8	post-occipital	Increased after BQ chewing (t = 4.525, FDR-corrected p = 0.006)	Increased in both healthy (t = 2.526, p = 0.019) and BQ-dependent groups (t = 4.017, p = 0.0005)
